# Notch3/VEGF-A axis is involved in TAT-mediated proliferation of pulmonary artery smooth muscle cells: Implications for HIV-associated PAH

**DOI:** 10.1038/s41420-018-0087-9

**Published:** 2018-08-06

**Authors:** Ming-Lei Guo, Yeon Hee Kook, Callen E. Shannon, Shilpa Buch

**Affiliations:** Department of Pharmacology and Experimental Neuroscience, 985880 Nebraska Medical Center, University of Nebraska Medical Center, Omaha, NE 68198 USA

## Abstract

The incidence of pulmonary arterial hypertension (PAH) is a significant co-morbidity observed in HIV (+) individuals. Pulmonary artery smooth muscle cells (PASMCs)—key components of the arterial vessel wall that regulate vessel diameter, demonstrate increased proliferation and hypertrophy in the lungs of HIV infected individuals, underscoring the role of these cells in the pathogenesis of HIV-associated PAH. While several pathways have been implicated in enhanced proliferation of PASMCs, detailed molecular mechanism(s) underlying HIV-associated PASMC proliferation still remain elusive. In the current study, we sought to investigate the effects HIV protein transactivator of transcription (TAT)-mediated proliferation on PASMCs. In agreement with earlier findings, our results also demonstrated TAT-mediated proliferation of human PASMCs. We identified activation of a novel Notch3 signaling pathway in TAT-mediated proliferation of PASMCs. Further validation of the Notch 3 pathway was demonstrated using both pharmacological (γ-secretase inhibitor, DAPT), as well as genetic approaches (Notch3 siRNA). Vascular endothelial growth factor A (VEGF-A) was identified as a novel downstream molecule that was induced following Notch activation. Findings from in vitro studies were further validated in archived simian immunodeficiency virus (SIV)-infected monkey lung tissues. There was increased activation of Notch3 signaling as well as enhanced expression of VEGF-A in the lungs of SIV-infected macaques compared with the lungs of SIV(−) controls. Taken together, we demonstrated that HIV-TAT increased the proliferation of PASMCs via the Notch3/VEGF-A axis. Targeting the Notch3/VEGF-A axis could thus be considered a potential therapeutic approach for the treatment of HIV-associated PAH.

## Introduction

In the era of combined anti-retroviral therapy (cART), HIV (+) infection has transitioned from a life-threatening disease into a chronic, manageable disease owing to efficient suppression of plasma viremia with viral loads often below the threshold of detection^1,2^. As HIV (+) individuals on cART continue to live longer, paradoxically, the prevalence of HIV-associated comorbidities is also on a rise. Examples of these comorbidities include HIV-associated neurocognitive disorders^3,4^, liver and kidney dysfunctions^5^, cardiovascular diseases^6^, and pulmonary arterial hypertension (PAH)^7,8^.

PAH is a subset of pulmonary hypertension disorders, which are histologically characterized by pulmonary arterial remodeling, formation of plexiform lesions, and poor clinical outcomes. Epidemiological studies have shown that the incidence of PAH in HIV(+) individuals is ~25-fold higher compared with that in HIV(−) subjects, indicating thereby a strong positive association between HIV infection and PAH^9,10^. Several previous studies have demonstrated that HIV infection coincided with the development of pulmonary vascular diseases, including severe PAH^11,12^. Attempts aimed at identifying mature HIV particles in the lung tissue however, have failed despite use of multiple technological approaches, such as electron microscopy, immunohistochemistry, DNA in situ hybridization, and polymerase chain reaction^13,14^. It is now generally accepted that HIV does not directly affect lung pathogenesis. The focus therefore has shifted to examining the contribution of HIV viral proteins in mediating the pathogenesis of HIV-associated PAH^15,16^. Additionally, it is now well recognized that viral proteins such as transactivator of transcription (TAT), viral envelope gp120, and Nef continue to persist despite undetectable viremia in the presence of cART^1,2^. Role of some of these proteins has been extensively investigated for their contribution to the pathogenesis of HIV-associated PAH. Nef is a critical protein implicated in HIV replication and pathogenesis and was found to contribute to the pulmonary pathology in simian immunodeficiency virus (SIV)-infected macaques^17^. Similarly, gp120 has been demonstrated to induce vascular smooth cell proliferation as well as endothelial cell apoptosis^18^. Notably, gp120 was shown to significantly increase the secretion of vasoconstrictor endothelin-1, a well-established molecule implicated in the pathogenesis of PAH, in human lung endothelial cells^19^. Another critical viral protein, TAT, has also been shown to enhance the development of PAH. TAT can be easily taken up by the uninfected cells including the lung vascular endothelium via multiple cellular mechanisms^20,21^. For example, TAT interacts with endothelial cells via cell membrane receptors, including integrins and the vascular endothelial growth factor receptor-2/kinase insert domain receptor^20,21^. Increased expression of interleukin (IL)-2 and the HIV-1 co-receptor CCR5 have also been to aid in the entry of TAT in the cells. TAT can also induce the expression of hypoxia-inducible factor-1α (HIF-1α) protein, as well as the smooth muscle cell mitogen, platelet-derived growth factor-B (PDGF-B) in endothelial cells^22^, both mediators that are critical for the initiation and sustenance of proliferation pathways, contributing to pulmonary vascular remodeling and the development of PAH^23^.

Besides endothelial cells, pulmonary artery smooth muscle cells (PASMCs) have also been garnering attention for their contribution in the pathogenesis of PAH. Multiple signaling pathways including alterations in microRNA expression, as well as activation of PDGF signaling have been suggested to underlie increased proliferation of PASMCs and progression to PAH^23–25^. TAT has been suggested to activate PDGF signaling pathways by increasing the production of reactive oxygen species (ROS) resulting in enhanced PASMCs proliferation^26^. Notch signaling is critical for modulating proliferation and differentiation of PASMCs^27,28^. Upregulated Notch ligands and Notch3 receptors in PASMCs have been reported to promote the development of pulmonary vascular remodeling in patients with PAH and in animals with experimental pulmonary hypertension (EPH)^29^. However, the possible role of Notch signaling in HIV-associated PAH has never been explored to date.

In the current study, we investigated the effects of TAT protein on the proliferation PASMCs and investigated the role of Notch signaling in this process. Our findings showed that TAT exposure increased proliferation of PASMCs by activing Notch3 signaling followed by increased expression of vascular endothelial growth factor A (VEGF-A). These results identify a previously unknown pathway involved in the pathogenesis of PAH in HIV (+) individuals, implying thereby that targeting this axis could be considered as a potential therapeutic approach for ameliorating HIV-associated PAH.

## Results

### HIV-TAT-mediated proliferation of human PASMCs

**Fig. 1:**
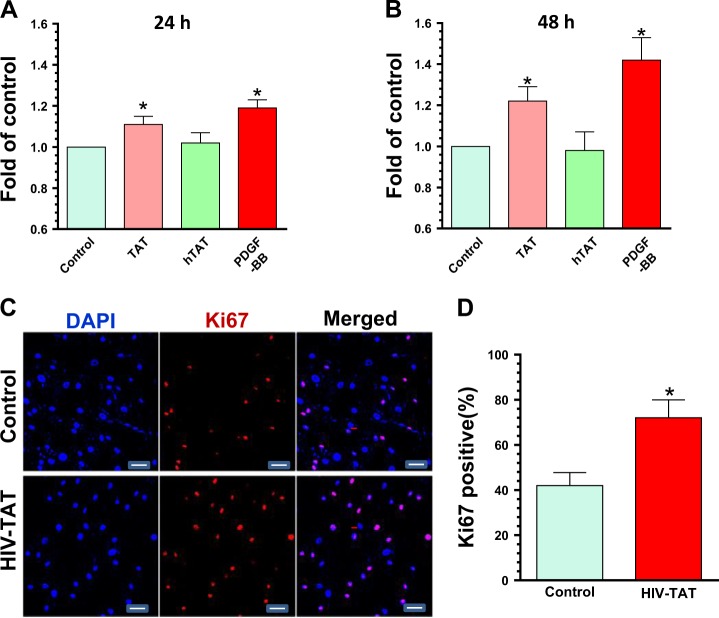
HIV-TAT mediated upregulatin of human PASMCs proliferation. PASMCs were seeded into 96-well plates and exposed to different reagents: control, TAT, heated TAT, and PDGF-BB. At the indicted time period, cells were subjected to the CyQuant cell proliferation assays. (**a**) TAT significantly increased PASMCs proliferation after 24 h exposure (*P* < 0.05). (**b**) TAT significantly increased PASMCs proliferation after 48 h exposure (*P* < 0.05). (**c** and **d**) TAT substantially increased the percentage of Ki67 positive cells compared to the control group. Scale bar = 5 µM. Statistical results were expressed as means ± SEM of four independent experiments and were analyzed using ANOVA (*P < 0.05 vs. control)

Epidemiological studies indicate that the incidence of PAH in HIV(+) individuals is as high as 25 fold compared with the HIV(−) controls^9,10^. Based on the fact that HIV is undetectable in the lungs of infected individuals, it is likely that HIV proteins, such as TAT and/or gp120 play critical roles in the pathogenesis of PAH^13,14^. PASMCs are a critical component of blood vessel walls and increased proliferation of these cells is a key contributor of PAH. Herein we explored the effects of TAT protein on SMC proliferation. Human PASMCs were exposed to TAT (200 ng/ml) for upto 48 h and cell numbers monitored using CyQuant cell proliferation assays. Platelet-derived growth factor-BB (PDGF-BB, 20 ng/ml), a known mitogen of SMC growth, was used as a positive control. Our results clearly demonstrated that TAT exposure of SMC significantly increased their proliferation at both 24 and 48 h (Fig. 1a, b, 1.11 ± 0.04 fold at 24 h; 1.23 ± 0.07 fold at 48 h, *P* < 0.05). Heated TAT (hTAT) on the other hand failed to exert any effect on SMC proliferation. As expected, PDGF-BB exhibited similar effects on SMC growth as that observed for TAT. To further validate our findings, we next sought to detect the expression of Ki67 (proliferation marker) in TAT exposed SMC. PASMCs were cultured on glass coverslips in 24-well plates and exposed to TAT (200 ng) for 24 h, followed by fixation and detection of Ki67 by fluorescent immunostaining. As expected, the percentage of Ki67-positive cells in the TAT exposed group was significantly higher than in control cells (72 ± 7.98% vs. 42 ± 5.81%, *P* < 0.05; Fig. 1c, d). Taken together, our findings demonstrated that exposure of SMC to HIV TAT has stimulated cell proliferation.

### HIV-TAT activated notch3-signaling in human PASMC

**Fig. 2:**
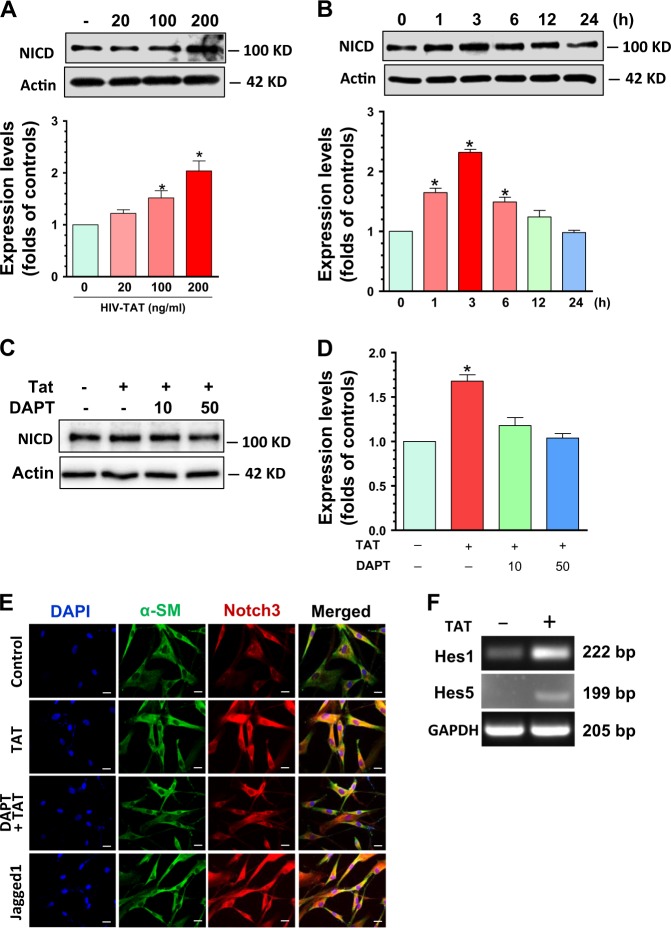
HIV-TAT induced Notch3 activation in human PASMCs. PASMCs were cultured and received different doses of TAT (20, 100, and 200 ng/ml) or 200 ng/ml for different time period (1–24 h). Cells were then collected and protein homogenates were prepared for assessment of NICD levels by WBs. **(a**) TAT increased NICD levels in a dose-dependent manner (*P* < 0.05). (**b)** TAT increased NICD levels in a time-dependent manner. **(c** and **d**) Pre-treatment of DAPT 1 h significantly blocked the upregulation effects of NICD induced by TAT (*P* < 0.05). (**e**) PASMCs were seeded into 24-well plates followed with indicated treatments. Then cells were immunostained with Notch3/α-SM antibody. The results showed that TAT increased Notch3 signal intensity and DAPT inhibited the upregulation of Notch3. Scale bar = 2 µM. **(f)** Cells were seeded into six-well plates receiving TAT exposure for 6 h. Then cells were collected for total RNA isolation. The mRNA levels of Hes1 and 5 were monitored by RT-PCR. GAPDH was used as an internal control. TAT significantly increased the mRNA levels of Hes1 and 5. Statistical results were expressed as means ± SEM of four independent experiments and were analyzed using ANOVA (*P < 0.05 vs. control)

Based on the fact that Notch3 signaling plays a role in SMC proliferation as well as in the development of PAH^27,28^ and, also since TAT can interact with Notch via the EGF-like domains^30^, we next sought to examine whether TAT exposure could lead to activation of Notch3 signaling to promote proliferation of SMC. To investigate the dose-dependent effects of TAT on Notch3 activation, human PASMCs were exposed to varying concentrations (20, 100, and 200 ng/ml) of TAT for 24 h followed by detection of Notch intracellular domain (NICD) levels by western blotting (WB). Our findings demonstrated that TAT significantly increased the levels of NICD at a concentration of both 100 and 200 ng/ml TAT, with a maximum effect at 200 ng/ml. TAT at a dose of 20 ng/ml failed to exhibit any significant upregulation of NICD (Fig. 2a, *P* < 0.05). We next determined the time-course of TAT effect on NICD expression. Cells were exposed to TAT (200 ng/ml) for varying time points (1–24 h) followed by assessment of NICD expression by WB. Our results clearly demonstrated that exposure of SMC to TAT resulted in increased expression of NICD starting at 1 h and this upregulation was sustained up to 6 h post-TAT treatment. NICD levels gradually declined to baseline after 6 h post treatment (Fig. 2b). We next investigated whether TAT-mediated activation of Notch3 activation could be blocked by the γ-secretase inhibitor DAPT, a well-known Notch pathway inhibitor^31^. Human PASMCs were pre-treated with DAPT (10 or 50 μmol/l) for 1 h followed by exposure to HIV-TAT for another 3 h, and assessed for expression of NICD by WBs. Our findings demonstrated that DAPT treatment significantly blocked TAT-mediated upregulation of NICD (Fig. 2c, d, *P* < 0.05). To further validate the blocking effects of DAPT on TAT-mediated activation, we performed double fluorescent immunostaining of α-SM (SMC marker) and Notch3. Notch3 ligand Jagged-1 was used as a positive control. As expected, TAT exposure resulted in upregulation and cytosolic accumulation of Notch3 and this effect was blocked in cells pre-treated with DAPT (Fig. 2e). The endpoint readout for Notch3 activation is increased transcription of Hes1 and Hes5 genes. We thus monitored the transcriptional expression of both Hes1 and Hes5 by real-time PCR in PASMC exposed to TAT. Our findings demonstrated that mRNA levels of both Hes1 and Hes5 were significantly upregulated in TAT exposed group compared with cells exposed to vehicle (Fig. 2f). Taken together, our findings suggest that TAT can activate Notch3 signaling in PASMCs.

### Activation of notch3 signaling is critical for TAT-mediated upregulation of VEGF-A levels

**Fig. 3:**
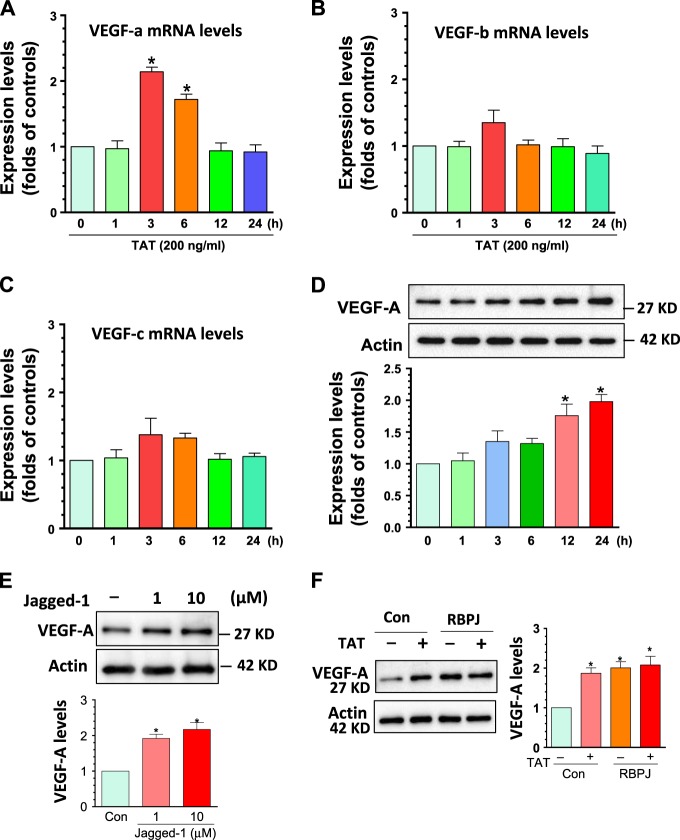
Notch3 activation was involved in TAT-mediated upregulation of VEGF-A. PASMCs were seeded into 24-well plates with TAT (200 ng/ml) exposure for different time periods (1–24 h). Then cells were collected for total RNA/protein extraction for monitoring the mRNA levels of VEGF-A, B and C and protein levels of VEGF-A. (**a)** TAT significantly increased the VEGF-a mRNA levels. **(b)** TAT exerted no effects on VEGF-B levels. (**c)** TAT exerted no effects on VEGF-C levels. **(d)** TAT significantly increased the VEGF-A protein levels. **(e)** PASMCs were exposed to Notch ligand Jagged-1 followed with the detection of VEGF-A levels. Jagged-1 significantly increased VEGF-A levels. (**f**) TAT failed to induce the expression of VEGF-A in RBPJ transfected cells. Representative WBs are shown and statistical results are expressed as means ± SEM of four independent experiments and were analyzed using ANOVA (*P < 0.05 vs. control)

VEGF members belong to a family of growth factors that play key role in inducing proliferation in various types of cells^32^. Previous studies have demonstrated that HIV infection leads to increased expression of VEGF in podocytes^33^. We thus sought to explore whether similar to HIV infection, HIV proteins could also regulate the expression of VEGF in human PASMCs. Cultured cells were exposed to TAT for varying time periods (1–24 h), followed by extraction of total RNA and assessment of the VEGF isoforms (A, B, and C) by quantitative (q) RT-PCR. Our findings showed that the levels of VEGF-A were significantly increased at 3 h post-TAT treatment and this effect persisted for upto 6 h, after which the levels gradually dropped to baseline (Fig. 3a). In contrast, the other two isoforms of VEGF (B and C) failed to demonstrate any significant change in mRNA expression, thereby indicating an isoform-specific effect of TAT on VEGF-A (Fig. 3b, c). We next investigated the expression of VEGF-A protein. Consistent with the mRNA expression, VEGF-A protein levels were also significantly upregulated in PASMCs exposed to TAT, albeit at later time points (*P* < 0.05, Fig. 3d). Additionally, we also assessed the direct effect of Notch3 activation in PASMCs in response to the Notch ligand Jagged-1 (1, 10 µM for 24 h). Our findings demonstrated that Jagged-1-induced significant induction of VEGF-A expression at both the tested concentrations (*P* < 0.05, Fig. 3e). To further explore the role of Notch3 in TAT-mediated effects on VEGF-A levels, PASMCs were transfected with either control (pcDNA) or RBPJ recombinant plasmids for 24 h followed by exposure to TAT for additional 24 h. RBPJ is a downstream transcriptional regulator which has been demonstrated to play a central role in Notch signaling activation and cell proliferation^34^. Following TAT exposure, cells were collected for detection of VEGF-A protein levels. As expected, TAT significantly increased VEGF-A levels, however, this effect of TAT was not observed in RBPJ transfected cells likely because VEGF-A was substantially upregulated in the presence of RBPJ overexpression and hence, TAT failed to exert any further effect (Fig. 3f). These results implicate that both TAT and RBPJ function to activate the Notch3 pathway. Taken together, our findings suggest that TAT can upregulate the expression of VEGF-A at both the mRNA and protein levels in PASMCs and that this involves activation of the Notch3 pathway.

### Notch3 inhibition blocked TAT-mediated upregulation of VEGF-A

**Fig. 4:**
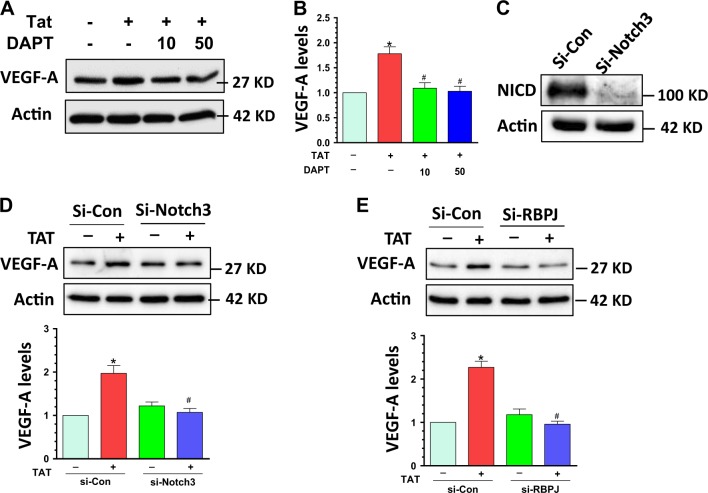
Notch3 inhibition ameliorated TAT-mediated induction of VEGF-A. PASMCs were pre-treated with DAPT followed by exposure of cells to TAT and assessment of expression of VEGF-A levels. **(a** and **b)** DAPT significantly blocked TAT-mediated upregulation VEGF-A levels (*P* < 0.05). **(c**) The levels of NICD were substantially inhibited in siRNA-Notch3 transfected cells. **(d**) SiRNA-Notch3 transfection significantly blocked TAT-mediated upregulation of VEGF-A levels (*P* < 0.05). **(e)** SiRNA-RBPJ transfected cells failed to exhibit TAT-mediated upregulation of VEGF-A levels (*P* < 0.05). Representative WBs are shown and statistical results are expressed as means ± SEM of four independent experiments and were analyzed using ANOVA (*P < 0.05 vs. control; #*P* < 0.05 vs. TAT-treated group)

Having shown that TAT-mediated activation of Notch3 resulted in elevated expression of VEGF-A, we next sought to explore the effect of blocking Notch3 on TAT-mediated expression of VEGF-A. Human PASMCs were pretreated with DAPT for 1 h followed by exposure to TAT exposure for 24 h and cells assessed for expression of VEGF-A protein levels. Our findings showed that DAPT pre-treatment significantly blocked TAT-mediated induction of VEGF-A expression (Fig. 4a, b, *P* < 0.05). These findings were further validated using a genetic approach wherein cells were transfected with either siRNA-Notch3/scrambled and then exposed to TAT and assessed for silencing of Notch3. As shown in Fig. 4c, Notch3 levels were significantly downregulated in siRNA-Notch3 transfected PASMCs but not in the scrambled siRNA-transfected cells. Furthermore, and as expected, transfection of siRNA-Notch3 significantly blocked TAT-mediated induction of VEGF-A (Fig. 4d). Further validation was also done in cells transfected with siRNA-RBPJ, wherein TAT failed to mediate the induction of VEGF-A expression. Taken together, our findings suggest that inhibition of Notch3 signaling blocked TAT mediated upregulation of the downstream target VEGF-A.

### Notch3-VEGF-A axis contributes to TAT-mediated induction of PASMC proliferation

**Fig. 5:**
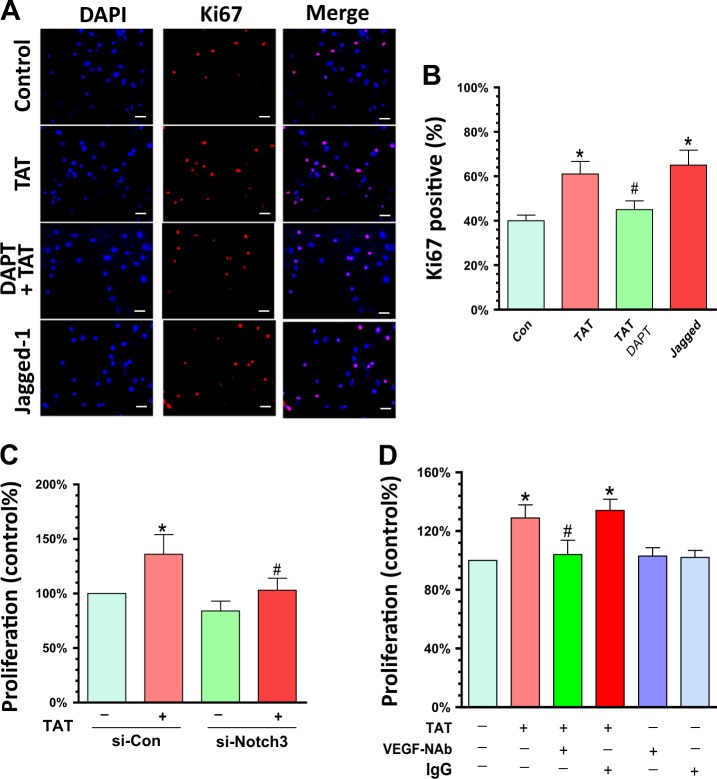
Activation of Notch3-VEGF axis contributes to TAT-mediated proliferation of human PASMC. **(a** and **b)** PASMCs were seeded into 24-well plates followed by the indicated treatments. Cells were then immunostained with Ki67 antibody. TAT significantly increased the percentage of Ki67 positive cells and pre-treatment with DAPT blocked this effect. Scale bar = 2.5 µM. (**c)** Cells were transfected with si-Con/Notch3 followed by exposure to TAT and cells assessed for proliferation using the CyQuant cell proliferation assays. DAPT pre-treatment significantly inhibited TAT-mediated proliferation in PASMCs (*P* < 0.05). (**d)** Cells were pre-incubated with VEGF-NAb followed by exposure to TAT and assessment of proliferation using the CyQuant cell proliferation assays. VEGF-NAb pre-incubation significantly blocked TAT-mediated proliferation of PASMCs (*P* < 0.05). Statistical results are expressed as means ± SEM of four independent experiments and were analyzed using ANOVA (*P < 0.05 vs. control; #*P* < 0.05 vs. TAT-treated group)

We next sought to determine the role of Notch3-VEGF-A axis in TAT-mediated proliferation of PASMCs. For this cells cultured on coverslips were pre-treated with DAPT for 1 h followed by exposure to TAT for an additional 24 h. Notch3 ligand Jagged-1 was used as positive control. Following treatment, cells were immunostained with the proliferation marker Ki67 and as shown in Fig. 5a, b, and consistent with the previous results, TAT significantly increased the percentage of Ki67-positive cells, and pre-treatment with DAPT blocked this effect (*P* < 0.05). As expected, Jagged-1 also significantly increased the number of Ki67 positive cells. We next utilized a genetic approach to investigate the role of Notch3 signaling in TAT-mediated proliferation of PASMCs. Cells were transfected with siRNA-Notch3/scrambled for 24 h followed by exposure of cells to TAT exposure for additional 24 h and assessed for proliferation using the CyQuant cell proliferation assays. As shown in Fig. 5c, TAT exposure resulted in significantly increased proliferation of PASMCs by ~63% compared with control cells not exposed to TAT (*P* < 0.05). In cells transfected with Notch3 siRNA there was a failure of TAT to induce proliferation. Next to examine the role of VEGF-A on TAT-mediated proliferation of PASMCs, cells were pre-treated with VEGF neutralizing antibody (NAb) for 1 h followed by exposure of cells to TAT for 24 h and assessed for proliferation. Our findings demonstrated that pretreatment with VEGF-NAb also ameliorated TAT-mediated induction of proliferation of PASMCs (Fig. 5d). These findings thus underscore the role of Notch3-VEGF axis in TAT-mediated proliferation of PASMCs.

### Increased proliferation in the lungs of SIV-infected macaques

**Fig. 6:**
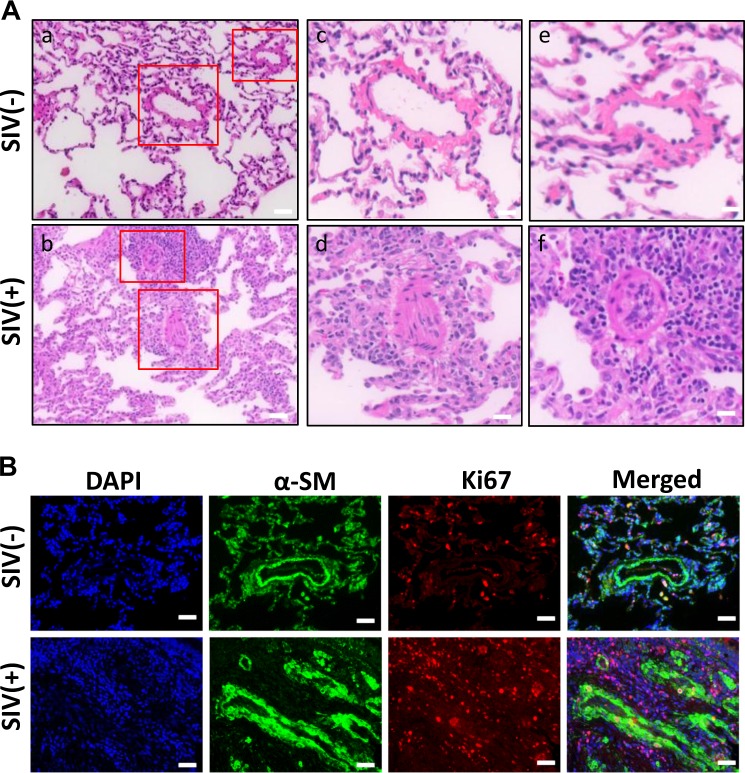
Increased proliferation in the lungs of SIV-infected macaques. **(a)** Hematoxylin and eosin staining demonstrating intima thickening and luminal stenosis in SIV-infected lungs compared with the lungs from uninfected macaques (*n* = 3). Scale bar in pictures (a, b) = 25 µM; scale bar in pictures (c–f) = 12.5 µM. **(b)** Ki67 immunostaining was performed to detect the proliferation cells in lung sections from SIV-infected macaques or saline-treated controls. Scale bar = 25 µM. There were significantly increased Ki67+ cells in SIV-infected lung tissues compared with control animals. Representative images are shown here

Having demonstrated that TAT promoted proliferation of human PASMCs in vitro, we next sought to explore whether we could recapitulate our results ex vivo. Lung tissues from archived SIV-infected (6 months) and uninfected rhesus macaques were utilized for this purpose. Hematoxylin and eosin staining was performed on lung sections from the two groups of macaques. As shown in Fig. 6a, there was significant intima thickening and luminal stenosis in SIV-infected lung tissue compared to uninfected lung tissue (Fig. 6a). The immunostaining results also revealed significantly increased Ki67+ cells around the blood vessels in SIV-infected lung tissues compared to the saline-treated controls (Fig. 6b).

### Upregulation of notch3 signaling in SIV-infected macaque lungs

**Fig. 7:**
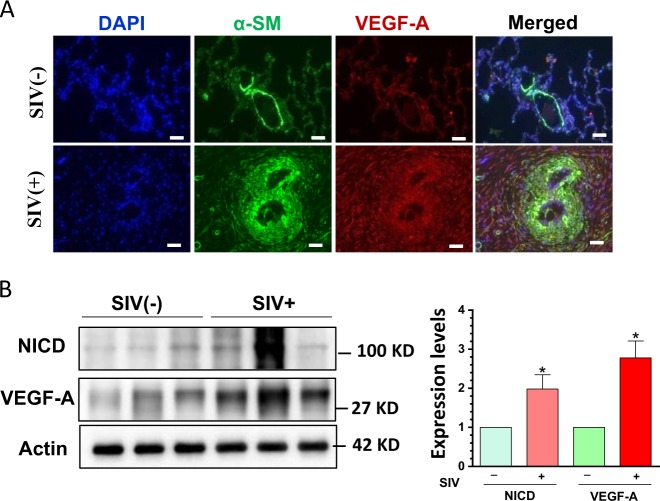
Upregulated levels of NICD and VEGF-A in the lungs of SIV-infected macaques. **(a)** Double immunostaining with α-SM and VEGF-A was performed on macaque lung sections with/without SIV infection. VEGF-A expression was significantly increased in SIV-infected lungs compared to the lungs from uninfected controls (*n* = 3). Scale bar = 10 µM. **(b)** Protein homogenates were extracted from lung tissues of both groups of macaques and were examined for the expression levels of NICD and VEGF-A. There was significantly increased expression of both NICD and VEGF-A in the lungs of SIV-infected vs. uninfected macaque lungs (*P* < 0.05). Representative WBs are shown and statistical results are expressed as means ± SEM of four independent experiments and were analyzed using ANOVA (*P < 0.05 vs. control; #*P* < 0.05 vs. TAT-treated group)

Next we sought to confirm activation of Notch3 signaling in the lungs of SIV-infected macaques. Double immunostaining for α-SM and VEGF-A was performed on monkey lung sections with/without SIV infection. As shown in Fig. 7a, VEGF-A levels were significantly increased in the SMC of SIV-infected lungs compared with the control animals. Protein homogenates were also extracted from lung tissues of the two groups of macaques and examine for the expression levels of NICD and VEGF-A. As expected, the expression levels for both NICD and VEGF-A were significantly increased in SIV-infected lung tissues (*P* < 0.05, Fig. 7b) compared with the lungs from control macaques.

## Discussion

**Fig. 8:**
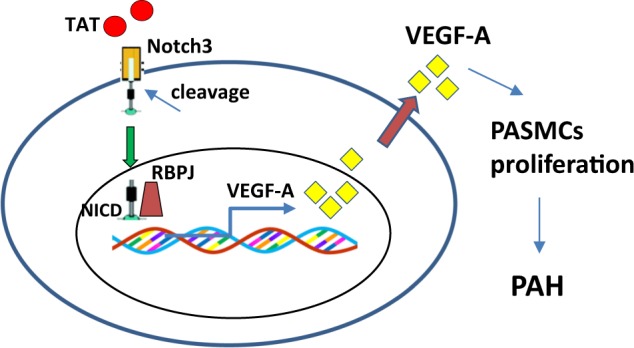
Schematic for TAT-mediated induction of HIV-associated PAH

HIV-associated PAH is a co-morbidity observed in HIV (+) individuals. HIV protein mediated increased proliferation of both the endothelium and SMCs is well-recognized as a causative factor contributing to the pathogenesis of PAH^7,8^. Although various pathways have been implicated in this process, the role of Notch signaling has never been explored. Our findings provide a novel pathway underlying TAT-mediated proliferation of PASMCs. In this study, we demonstrate that TAT-mediated activation of Notch3 signaling resulting in elevated expression of VEGF-A levels, leads to enhanced proliferation of PASMCs, thereby contributing to PAH pathogenesis. A schematic is presented in Fig. 8.

Notch signaling plays a role in multiple facets of blood vessel biology, such as vascular development, cell proliferation, and differentiation, as well as vascular remodeling^35–38^. Four Notch receptors (Notch1–4) and five Notch ligands including Jagged (Jag-1/2) and Delta-like (Dll1, 3, 4) have been identified thus far, with differential expression patterns depending on the cell types^36^. Of note, Notch3 has been demonstrated to be highly expressed in PASMCs^29^. Upon ligand binding, Notch receptors undergo several proteolytic processes that leads to the generation and release of NICD into the cytosol by the γ-secretase. Following this NICD translocates into the nucleus and binds with the transcription factor recombination signal binding protein for immunoglobulin-κ J region (RBPJ). The NICD/RBPJ complex, in turn, activates the downstream target genes including the transcriptional repressor hairy and enhancer of split (HES)^27,28,37^. Notch signaling has been demonstrated to regulate proliferation and differentiation of both endothelial cells and smooth muscle cells^35,36^. In animals with EPH, Notch3 activation has been shown to promote pulmonary vascular remodeling^29^. Consistent with these findings, inhibition of Notch signaling using the γ-secretase inhibitor DAPT, attenuated the development of vascular remodeling and reversed the progression of EPH^29^. HIV protein TAT has been suggested to interact with the EGF-like repeats of the Notch proteins^30^ however, the consequence of this interaction between TAT and Notch remains elusive. Our results demonstrated that TAT can significantly increase the expression of NICD indicating thereby that TAT can activate Notch3 signaling, which was also confirmed by assessing the expression levels of the downstream signature genes Hes-1 and 5. Role of Notch 3 was further confirmed by treating the cells with the γ-secretase inhibitor DAPT, which blocked TAT-mediated upregulation of NICD and ensuing PASMC proliferation. In addition to pharmacological approach, genetic silencing of Notch3 activity by transfecting the cells with Notch3/RBPJ siRNAs also resulted in amelioration of TAT-mediated upregulation of VEGF-A expression, thereby further underpinning the role of Notch3 activation in TAT-mediated proliferation of PASMCs. TAT has been suggested to increase endothelial cell proliferation by interaction with multiple pathways, such as the Ras/ERK MAPK signaling^39^, integrin αβ3-mediated pathway^40^, and the endothelial nitric oxide-induced pathway^41^. In addition to endothelial cells, TAT can also mediate PASMCs proliferation via alternative pathways including the bone morphogenetic protein receptor axis^42^, redox-related signaling pathways^26^, and Hes1/p27Kip1 signaling^43^. Our results demonstrated TAT can directly activate Notch3 signaling axis leading to the development HIV-associated PAH. Our findings on TAT-mediated activation of Notch3 signaling are in agreement with a recent publication of Fan et al. that has also demonstrated the role of TAT as a Notch ligand in impaired neurogenesis^44^. In this study it was shown that TAT inhibited neural progenitor cells (NPC) proliferation and migration and altered NPC differentiation and that immunodepletion of TAT from TAT-containing conditioned media or treatment of cells with heat inactivated TAT abrogated those effects. Furthermore, it was also shown that treatment with the γ-secretase inhibitor DAPT, rescued Tat-mediated impairment of NPC differentiation in vitro as well as neurogenesis in vivo. Currently, it remains unclear whether the interaction between TAT and Notch protein is a direct one or an indirect one and warrants further investigation.

Another significance of our findings is that we identified VEGF-A as a novel downstream effector of Notch3 activation. Translocation of NICD into the nucleus following exposure with either TAT or the Notch ligand Jagged-1, resulted in upregulation of VEGF-A mRNA and protein levels. Correspondingly, pretreatment of cells with the inhibitor—DAPT, resulted in amelioration of TAT-mediated upregulation of VEGF-A levels. Furthermore, pretreatment of cells with VEGF NAb also inhibited TAT-mediated proliferation of PASMC, thereby underscoring the critical role of the Notch3/VEGF-A axis in this process. Notch signaling and the VEGF pathway have been suggested to play a key role in vascular development and often involve bidirectional signaling, leading in turn, to induction of common downstream components^45,46^. For instance, while VEGF is a target of Notch signaling, activation of VEGF signaling can also lead to activation of Notch signaling^47^. Reciprocally, activation of Notch pathway can also regulate multiple angiogenic pathways including the expression of VEGF receptors^48^. Whether the NICD/RBPJ complex can directly bind with the promotor of VEGF deserves further exploration.

The non-human primate model of SIV-infected monkey recapitulates multiple facets of HIV infection in humans^7,8^. SIV infection results in histopathologic changes in pulmonary arteries that are characteristic of human HIV-associated PAH. Previous studies have shown that pulmonary blood vessels are adversely affected in SIV-infected monkeys, whereas the vasculature of other parenchymal organs are less frequently involved^14^. The pulmonary arteriopathy in macaques was characterized by abnormal pulmonary endothelial cells and plexiform lesions including medial proliferation, hypertrophy, and perivascular inflammatory cell deposition^17,49^. Our findings obtained from archival macaque lung tissues is in agreement with previous findings^42^. Interestingly, we have demonstrated activation of Notch3 signaling and elevated expression of VEGF-A in the lungs of SIV-infected macaques and this correlated with increased proliferation around the blood vessels. Take together our findings implicate dysregulated Notch3/VEGF axis as a contributing factor in the pathogenesis of HIV-associated PAH.

## Materials and methods

### Reagents

Recombinant Tat101 was purchased from ImmunoDiagnostics. Recombinant PDGF-BB was purchased from R&D Systems. Jagged-1 and DAPT were purchased from Sigma. Antibodies were obtained from the following sources: Ki67 (abcam), NICD (cell signaling), Actin (sigma), α-SM, Notch3 (cell signaling), VEGF-A (abcam); goat anti-rabbit (sc-2004) and goat anti-mouse (sc-2005) were from Santa Cruz Biotechnology. Flag-tagged CSL-VP16 plasmids were obtained from Dr. Aly Karsan (University of British Columbia, Vancouver, Canada) and control *Notch3 siRNA* (sc-29798), *RBPJ siRNA* (sc-41446), and scrambled *siRNA* (sc-37007) were from Santa Cruz Biotechnology.

### Human pulmonary artery smooth muscle cell culture (HPASMCs)

HPASMCs were purchased from ScienCell, and are routinely used and maintained in our lab. The cells are maintained in smooth muscle cell medium with 2% smooth muscle cell growth supplement and 1% antibiotic at 37 °C and 5% CO_2_ and used under passage number 14.

### SiRNA/plasmid transfection

HPASMCs were seeded into six-well plates to grow to 80% confluence. The next day, individual targeted siRNAs and scrambled siRNA (30 pM) or plasmids were mixed with lipofectamine 2000 (2 µl) in 100 µl opti-MEM (life technology, 31985062) and following 20 min incubation at room temperature, mixed liquids were dropped into cell culture medium (serum free) and incubated for additional 6 h. The medium was then changed to 2% FBS-containing medium for 20 h incubation, following which the transfected cells were then ready for the following experiments.

### Western blotting

Treated cells were lysed using the Mammalian Cell Lysis kit (Sigma, MCL1-1KT). Equal amounts of the proteins were electrophoresed in a sodium dodecyl sulfate-polyacrylamide gel (12%) under reducing conditions followed by transfer to PVDF membranes (Millipore, IPVH00010). The blots were blocked with 5% nonfat dry milk in phosphate-buffered saline (PBS; 137 mM NaCl; 2.7 mM KCl; 10 mM Na_2_HPO_4_; 2 mM KH_2_PO_4_). The western blots were then probed with antibodies recognizing the indicated proteins. The protein amounts loaded were normalized according to the ACTB/β-actin signal, using an anti-ACTB antibody (Sigma, A5441). The secondary antibodies were HRP conjugated to goat anti-mouse/rabbit IgG (Santa Cruz, sc-2005 and sc-2004).

### Immunocytochemistry

For immunocytochemistry, HPASMCs were plated on coverslips. The next day cells were fixed with 4% paraformaldehyde for 15 min at room temperature, followed by permeabilization with 0.3% Triton X-100 (Fisher scientific, BP151-1) in PBS. Cells were then incubated with a blocking buffer containing 10% NGS in PBS for 1 h at room temperature followed by addition of targeted antibodies and incubated overnight at 4 °C. Finally, the secondary Alexa Fluor 488 goat anti-rabbit or anti-mouse IgG (Invitrogen, A-11008) were added at a 1:2000 dilution for 2 h. Cells were washed three times in PBS and mounted with prolong gold antifade reagent with 4,6-diamidino-2-phenylindole (Invitrogen, 36935).

### Immunohistochmeistry

Formalin-fixed, paraffin-embedded lung sections (5 μm) from SIV-infected monkeys or saline-treated control were used. After staining with indicated antibodies overnight, sections were washed three times in PBS followed by incubation in Alexa Fluor 594-conjugated anti-mouse (Life Technologies; catalog #A11005) and Alexa Fluor 488-conjugated anti-rabbit IgG (Life Technologies; catalog #A11008) for 2 h at RT. After a final washing with PBS, sections were mounted with the mounting medium (Prolong Gold Anti-fade Reagent; Invitrogen). Fluorescent images were acquired at RT on a Zeiss Observer using a Z1 inverted microscope with a (×40, numerical aperture 0.3) oil-immersion objective. Images were processed using the AxioVs40 Version 4.8.0.0 software (Carl Zeiss). Photographs were acquired with an AxioCam MRm digital camera and were analyzed with NIH ImageJ software (ImageJ; RRID: nif-0000-30467).

### RNA extraction, reverse transcription, and quantitative polymerase chain reaction (qPCR)

Total RNA was extracted using Trizol reagent (Invitrogen, 15596-018). Briefly, monolayer cells in six-well plates were washed with PBS and lysed directly adding 1 ml Trizol. Cell lysate was aspirated into new 1.5 ml microcentrifuge tubes and 0.2 ml of chloroform was added. After extensive vortexing, the samples were centrifuged at 10,000×*g* for 15 min at 4 °C. The upper aqueous phase was transferred to a new tube and 500 µl isopropyl alcohol was added. Samples were incubated for 10 min and centrifuged again to precipitate total RNA. Total RNA was dissolved in DEPC-treated H_2_O and quantified. Reverse transcription reactions were performed using a Verso cDNA kit (Invitrogen, AB-1453/B). The reaction system (20 µl) included 4 µl 5X cDNA synthesis buffer, 2 µl dNTP mix, 1 µl RNA primer, 1 µl RT enhancer, 1 µl Verso enzyme Mix (Invitrogen, AB-1453/B), total RNA template 1 µg, and a variable volume of water. Reaction conditions were set at 42 °C for 30 min. QPCRs were performed by using SYBR Green ROX qPCR Mastermix (Qiagen, 330510). Reaction systems were set up as follows: 10 µl SYBR Green Mastermix, 0.5 µl forward primers, 0.5 µl reverse primers, and 9 µl distilled H_2_O. 96-well plates were placed into a 7500 fast real-time PCR system (Applied Biosystems, Grand Island, NY) for program running. Primers for human *VEGF-a, b, c* were commercial available from Qiagen company. Hes1 primer: forward 5′-CGGACATTCTGGAAATGACA-3′ and reverse 5′-TTGATCTGGGTCATGCAGTT-3′; Hese5 primer sequence: forward 5′-ACTCCAAGCTGGAGAAGGC-3′ and reverse 5′-GAAGTGGTACAGCAGCTTCATC-3′.

### CyQUANTcell proliferation assay

HPASMCs were seeded in 96-well plates at a density of 10^4^ cells/well and were serum starved for 16 h. Cells were then pre-treated with DAPT for 1 h followed by subsequent treatment with Tat for 24 h or 48 h. About 100 μl of Quant^®^ Direct reagent was added into each well and incubated in the CO_2_ incubator for 30 min. Fluorescence intensity of each well was obtained using a Dynatech MR5000 plate counter at excitation and emission wavelengths of 480 and 520 nm, respectively.

### Statistics

The results are presented as means ± SEM, and were evaluated using a one-way analysis of variance followed by a Bonferroni (Dunn) comparison of groups using least-squares-adjusted means. Probability levels of <0.05 were considered statistically significant.
